# Feature-Tracking-Derived Strain Analysis for Identification of Subendocardium-Involved Late Gadolinium Enhancement in Load-Induced Left Ventricular Hypertrophy: A Multicenter Study of Cardiac Magnetic Resonance Data

**DOI:** 10.3390/jcm12247543

**Published:** 2023-12-07

**Authors:** Ying Zhong, Qian Long, Mu Zeng, Lianming Wu, Liang Guo, Guan Wang

**Affiliations:** 1 Department of Radiology, The First Hospital of China Medical University, Shenyang 110001, China; zhongying@cmu.edu.cn; 2 Department of Radiology, The Second Xiangya Hospital, Central South University, Changsha 410011, China; longqian1990@csu.edu.cn (Q.L.); zengmu@csu.edu.cn (M.Z.); 3 National Clinical Research Center for Metabolic Diseases, Department of Metabolism and Endocrinology, The Second Xiangya Hospital, Central South University, Changsha 410011, China; 4 Department of Radiology, Renji Hospital, School of Medicine, Shanghai Jiao Tong University, Shanghai 200001, China; 5 Department of Cardiovascular Medicine, The First Hospital of China Medical University, Shenyang 110001, China

**Keywords:** load-induced LVH, SILGE, CMR-FT, strain analysis

## Abstract

Background: Subendocardium-involved late gadolinium enhancement (SILGE) is a significant predictor of poor prognosis in patients with load-induced left ventricular hypertrophy (LVH). Objectives: This multicenter study aimed to investigate whether the diagnostic performance of cardiac magnetic resonance feature-tracking (CMR-FT)-derived strain analysis for detecting subtle subendocardial injury would be influenced by its load dependence in patients with load-induced LVH. Methods: A total of 149 patients with load-induced LVH were recruited from three centers and underwent enhanced CMR imaging. The patients were divided into two groups based on the presence or absence of SILGE on CMR (SILGE^+^ group: *n* = 56; SILGE^−^ group: *n* = 93). Clinical and CMR parameters were evaluated in both groups. Results: The LV systolic pressure (LVSP) and LV end-diastolic pressure (LVEDP) in the SILGE^+^ group were higher than those in the SILGE^−^ group (each with *p* < 0.05), and LVSP and LVEDP were correlated with the LV global longitudinal strain (GLS) (each with *p* < 0.05) in research center 1. The LV strain parameters were significantly lower in the SILGE^+^ group than those in the SILGE^−^ group (each with *p* < 0.05). Logistic regression analysis identified GLS (OR 1.325; 95% CI 1.180 to 1.487, *p* < 0.001) as a predictive factor of SILGE in the patients with load-induced LVH. The receiver operating characteristic (ROC) curve analysis results indicated that the areas under the curve (AUC) of global radial strain (GRS), global circumferential strain (GCS), and GLS were 0.68, 0.69, and 0.76, respectively. De Long’s test results implied that GLS had the best diagnostic performance for SILGE (*p* = 0.04). Conclusion: Despite the load dependency of CMR-FT-derived strain analysis, the GLS exhibits reasonable accuracy in the identification of SILGE and can potentially serve as a feasible alternative for detecting subendocardial involvement in patients with load-induced LVH who are contraindicated for LGE.

## 1. Introduction

Load-induced left ventricular hypertrophy (LVH) is an abnormal increase in the LV myocardial mass caused by a chronically increased afterload, most commonly occurring in cases of hypertensive heart disease (HHD) and aortic stenosis (AS). Over time, without timeous and effective intervention, such patients transition from LVH to decompensation, and eventually develop heart failure [1,2].

The previous studies have shown that the presence of subendocardial infarct-like late gadolinium enhancement (LGE) in patients with LVH, but without significant coronary stenosis, is a significant predictor of heart failure and poor prognosis [3,4]. This subendocardium-involved LGE (SILGE) [5] may suggest the presence of unrecognized infarcts, which can arise from prolonged ischemia due to an imbalance between the oxygen supply and demand [6]. The early detection of the subtle subendocardial injury is clinically significant in wave-front ischemic progression. However, patients with load-induced LVH exhibit a notable prevalence of chronic kidney dysfunction [7,8], which presents challenges to their use of gadolinium-based contrast agents.

In recent years, previous studies have explored the potential clinical utility of the measurement of strain and the strain rate derived from using cardiac magnetic resonance feature tracking (CMR-FT) technology in detecting irreversible myocardial injuries [9]. However, the measurements of LV strains are sensitive to acute changes in the loads, which have been well documented both in animal models and human subjects [10,11]. It is unclear whether the diagnostic performance of CMR-FT-derived strain analysis would be affected by its load dependence in overload-induced LVH, especially for the subtle injury located in the subendocardium. The objective of this study was to estimate the diagnostic performance of CMR-FT-derived strain analysis for identifying SILGE in load-induced LVH in a multicenter setting.

## 2. Materials and Methods

### 2.1. Study Cohort

A total of 149 patients (66 patients with AS, and 83 patients with HHD) were recruited from three different medical centers (The First Hospital of China Medical University; Renji Hospital, School of Medicine, Shanghai Jiao Tong University; Xiangya Hospital of Central South University) for a CMR study between June 2019 and April 2023. The inclusion criteria for patients with load-induced LVH: AS, all patients referred for CMR in this time period had moderate or severe AS (based on the Doppler echocardiographic demonstration of mean aortic valve pressure gradient and peak transvalvular velocity, according to the American College of Cardiology/American Heart Association criteria [12]). HHD: patients were eligible for the study if they had a history of hypertension or evidence of LVH using any imaging modality. Individuals with a systolic blood pressure of more than 140 mmHg or a diastolic blood pressure of more than 90 mmHg on at least two separate occasions, or taking one or more medications for hypertension were included. Individuals with LVH were defined as having an LV mass index (LVMI) by body surface area, measured using CMR, of more than 81 g/m^2^ for men, or more than 61 g/m^2^ for women [13]. The exclusion criteria included other valve diseases, significant coronary artery stenosis (≥50%) on invasive coronary angiography (CAG) or coronary computed tomography angiography (CCTA), hypertrophic cardiomyopathy, amyloidosis, contraindications with CMR (including pacemaker and defibrillator implantation), and an estimated glomerular filtration rate (Cockcroft Gault equation) of <30 mL/min. The patients could be divided into the SILGE^+^ group (*n* = 56) or the SILGE^−^ group (*n* = 93) according to whether there was SILGE in the CMR images. The study was conducted according to the guidelines of the Declaration of Helsinki and approved by the Ethics Committee of The First Hospital of China Medical University (protocol code, 2023-394-2, and date of approval, 10 March 2023).

### 2.2. CMR Imaging

The CMR studies were conducted using 3.0T clinical magnetic resonance systems (MAGNETOM Verio and Skyra systems, Siemens Healthineers, Erlangen, Germany; Philips Healthcare, Best, The Netherlands) and a standardized protocol with stable study parameters. The specific scanning sequence and parameters are as follows: (1) Siemens: Steady-state free precession (SSFP) sequence (repetition time (TR)/echo time (TE): 41.3/1.5 ms; flip angle (FA): 50°; slice thickness (ST): 8 mm; field of view (FOV): 300 × 340 mm) was used to obtain cine images. An inversion recovery sequence with phase-sensitive inversion recovery sequences (PSIR) (TR/TE: 750/1.6 ms; FA: 40°; FOV: 320 × 350 mm) was used for obtaining late gadolinium enhancement (LGE) images. After 0.2 mmol/kg gadopentetate dimeglumine was injected, the LGE images were obtained within 8–15 min. (2): Philips: Balanced turbo field echo (BTFE) sequence (TR/TE: 2.9/1.7 ms; FA: 60°; ST: 7 mm; FOV: 300 × 300 mm) was adopted to obtain short-axis and long-axis cine images. Mid-diastolic inversion prepared a 2D gradient echo sequence (TR/TE: 3.3/1.7 ms; FA: 25°; FOV: 300 mm × 300 mm) to obtain LGE images.

### 2.3. Image Analysis and Post-Processing

The images were analyzed by operators from research center 1 with more than 3 years of experience, who were blinded to the clinical data, using CVI42 software (Circle Cardiovascular Imaging 42, Version 5.10.1, Calgary, AB, Canada) [14]. We plotted the contour of the LV endo and epicardial borders at the end of diastole and end of systole on the short-axis cine images to obtain the LV function, volume, and mass index parameters. We drew the contour of the LV endo and epicardial borders at the end of diastole on the short- and long-axes cine images to obtain the LV strain and strain rate.

For intra-observer reproducibility and inter-observer reproducibility, the observers measured the LV strains of 20 randomly selected cases from the patient cohort, from each research group (*n* = 60). For intra-observer reproducibility, one observer re-measured their LV strains 3 months later, blinded to the previous measurements. For inter-observer reproducibility, another observer measured the LV strains independently.

### 2.4. Measurement of LV Systolic Pressure (LVSP) and LV End-Diastolic Pressure (LVEDP)

In the present study, the LVSP of AS patients was the sum of the average transvalvular gradient of the aortic valve measured using transthoracic echocardiography and the arterial systolic blood pressure [15]. The LVSP of HHD patients was considered approximately equivalent to the radial arterial systolic pressure.

The LVEDP in the HHD patients was estimated using measurements of early mitral valve peak velocity (*E*) and peak early diastolic mitral annular velocity (*e*′) obtained using transthoracic echocardiography. Thus, the LVEDP estimated using echocardiography was calculated as 11.96 + 0.596*E*/*e*′ [16].

### 2.5. Statistical Analysis

SPSS 26.0 (IBM, Armonk, NY, USA) and MedCalc (Version 19.0.4, Ostend, Belgium) statistical software were used for statistical analysis. According to whether the statistics could meet the normal distribution, the independent sample *t*-test or Wilcoxon rank sum test was selected for comparison among the independent samples; continuous variables are expressed as mean ± standard deviation/median (interquartile range, IOR). The classification variables were compared using a *χ*^2^ test. Correlation analysis was performed to assess the association between the LV strains and left ventricular pressure (LVP) and N-terminal pro-brain natriuretic peptide (NT-proBNP). Univariable and multivariable logistic regression analysis was conducted to explore potential determinants of SILGE. Variables with univariable *p* < 0.10 were selected for multivariable analysis and are expressed as hazard ratios with 95% CIs. Receiver operating characteristic (ROC) curves were analyzed to determine the cut-off value of the continuous variables that predict SILGE. De Long’s test was performed to compare the differences in the AUC of strain parameters. Inter- and intra-observer analyses were conducted using intra-class correlation coefficients (ICCs). An ICC exceeding 0.75 was considered to indicate high consistency. A two-tailed *p* value of less than 0.05 was deemed statistically significant.

## 3. Results

### 3.1. Baseline Demographic Characteristics

**Figure 1:**
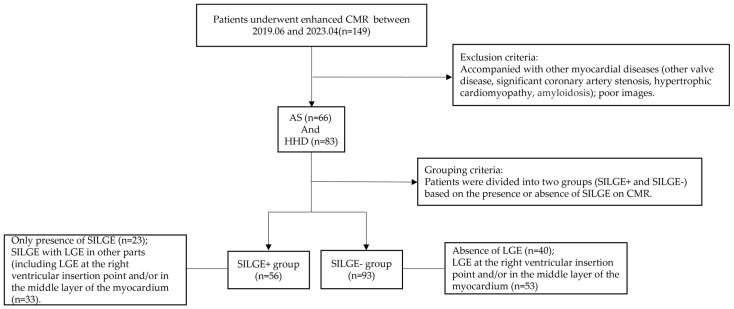
Study flow diagram showing the selection and group of patients. SILGE is shown in Figure 2. CMR, cardiac magnetic resonance; HHD, hypertensive heart disease; AS, aortic stenosis; SILGE, subendocardium-involved late gadolinium enhancement.

**Figure 2:**
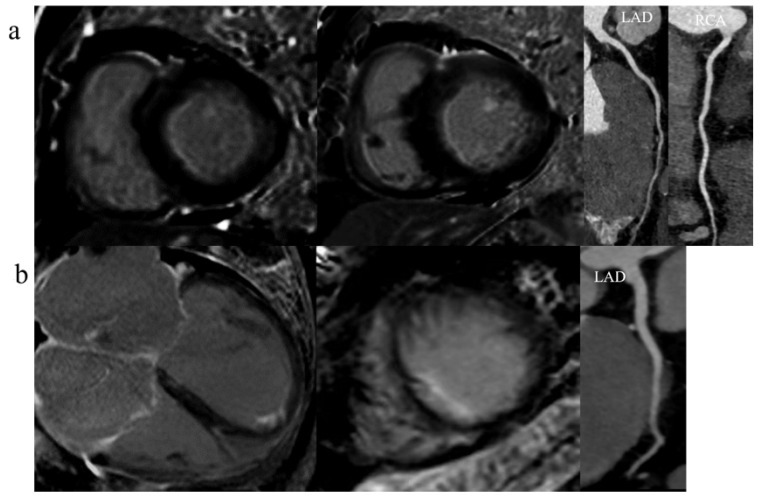
Types of SILGE: subendocardial linear LGE (**a**); subendocardial patchy LGE (**b**) in representative cases with normalized coronary artery confirmed with invasive CCTA. CCTA, coronary computed tomography angiography.

Some 149 patients were included in this study (Figure 1 shows the flow chart used to include/exclude patients). The patients were divided into the SILGE^+^ group (*n* = 56) and the SILGE^−^ group (*n* = 93), according to the presence or absence of SILGE (Figure 2). As shown in the flow chart, the SILGE^+^ group comprised SILGE without (*n* = 23) or with LGE in other regions (including LGE in the right ventricular insertion point, or in the middle layer of the myocardium, or both; *n* = 33), while the SILGE^−^ group consisted of non-LGE (*n* = 40) and LGE in other regions (including LGE in the right ventricular insertion point and/or in the middle layer of the myocardium; *n* = 53). Table 1 lists the basic clinical characteristics of all the patients in the two groups.

### 3.2. CMR Parameters of LV Remodeling and Function

**Figure 3:**
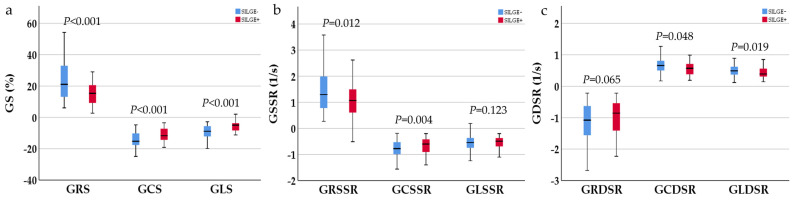
Clustered boxplot shows comparison of GS (**a**), GSSR (**b**), and GDSR (**c**) parameters in load-induced cardiac hypertrophy patients with and without SILGE. The detailed values are shown in Table 1. GS, global strain; GSSR, global systolic strain rate; GDSR, global diastolic strain rate.

For the whole study, detailed CMR parameters of the two groups are listed in Table 1. The GRS, GCS, GLS, global radial systolic strain rate (GRSSR), global circumferential systolic strain rate (GCSSR), global circumferential diastolic strain rate (GCDSR), and global longitudinal diastolic strain rate (GLDSR) in the SILGE^+^ group were lower than those in the SILGE^−^ group (Figure 3a–c), and the difference was statistically significant (each with *p* < 0.05). There was no significant difference in the left ventricular end-diastolic volume (LVEDV), left ventricular end-systolic volume (LVESV), left ventricular stroke volume (LVSV), left ventricular ejection fraction (LVEF), left ventricular mass index (LVMI), global longitudinal systolic strain rate (GLSSR), or global radial diastolic strain rate (GRDSR) between the SILGE^+^ and SILGE^−^ groups (each with *p* > 0.05).

### 3.3. NT-proBNP and CMR-FT-Derived Strain Parameters

**Figure 4:**
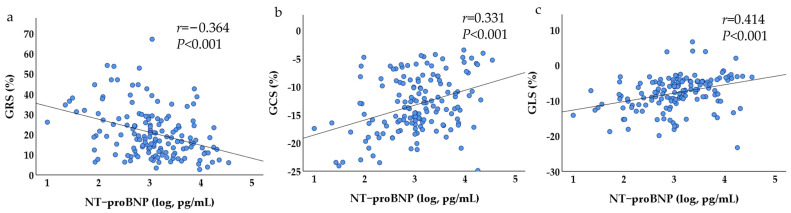
Scatter plots of correlation analysis between strain parameters and NT-proBNP. As NT-proBNP increases, the strains ((**a**) GRS, (**b**) GCS, and (**c**) GLS) gradually reduce.

NT-proBNP in the SILGE^+^ group was significantly higher than that in the SILGE^−^ group (1651.0 (2873.4) vs. 956.5 (2029.4), *p* < 0.05, Table 1). Correlation analysis showed that NT-proBNP had a good correlation with the strain parameters (GRS: *r* = −0.364; GCS: *r* = 0.331; GLS: *r* = 0.414; all with *p* < 0.001, Figure 4).

### 3.4. LVP in Load-Induced LVH Patients with and without SILGE in Research Center 1

In research center 1 in this study, 73 patients had LVSP and LVEDP parameters, with 33 patients in the SILGE^+^ group, and 40 patients in the SILGE^−^ group. Supplementary Table S1 describes the LVP of the two groups of patients. The LVSP and LVEDP were higher in the SILGE^+^ group than those in the SILGE^−^ group (LVSP: 175 ± 25 vs. 163 ± 22; LVEDP: 20 (5) vs. 20 ± 3, each *p* < 0.05). Correlation analysis showed that the GLS correlated with the LVSP (*r* = 0.237, *p* < 0.05) and LVEDP (*r* = 0.334, *p* < 0.005) (Supplementary Table S2).

### 3.5. Logistic Regression Model for Predicting SILGE

A logistic regression model for SILGE is shown in Table 2. The multivariable logistic regression model implies that the GLS (OR 1.325, 95%CI 1.180 to 1.487, *p* < 0.001) is an independent predictor of SILGE.

### 3.6. ROC Curve Analysis of LV Strains for Discriminating SILGE

**Figure 5:**
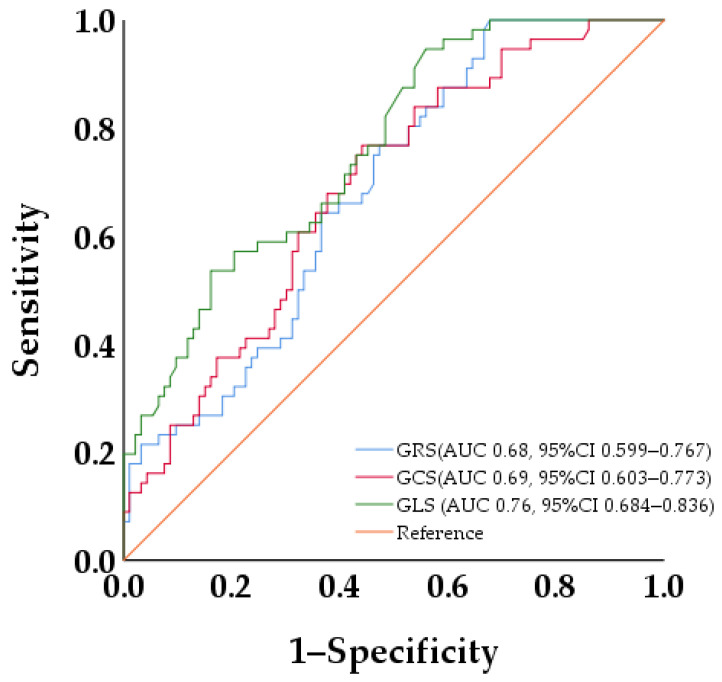
ROC analysis of strains for discriminating SILGE. ROC analysis showed best diagnostic performance of GLS for detecting SILGE.

The ROC curve analysis results indicated that the areas under the curve (AUC) of GRS, GCS, and GLS were 0.68, 0.69, and 0.76, respectively. De Long’s test results demonstrated that GLS had the best diagnostic performance for SILGE (0.68 vs. 0.69, *p* = 0.084; 0.68 vs. 0.76, *p* = 0.04; 0.69 vs. 0.76, *p* = 0.04) (Figure 5).

### 3.7. Inter- and Intra-Observer Reproducibility of CMR-FT-Derived Strain Parameters

As shown in Table 3, the LV strain parameters exhibited good reproducibility throughout the whole study cohort. The inter-research reproducibility was also excellent between the central reader (observer A) and the three different sites (Overall (*n* = 60): research center 1 (*n* = 20), research center 2 (*n* = 20), and research center 3 (*n* = 20)).

## 4. Discussion

Based on the previous reports demonstrating the feasibility of using the CMR-FT-derived strain for detecting MIs, the diagnostic capacity of the approach for subtle subendocardial injuries in load-induced LVH was evaluated. The results show that strain analysis can detect SILGE with reasonable accuracy; the GLS yielded the best AUC of all the strain parameters obtained. Importantly, our findings provided multicenter evidence that the CMR-FT-derived GLS is a viable alternative to LGE for detecting subendocardial involvement without the need for contrast agents.

In load-induced LVH, an excess intra-cavitary pressure and decreased vascular density due to myocardial hypertrophy may cause the impairment of myocardial blood flow, especially in the subendocardial region. The wave-front phenomenon of ischemic progression underlines the importance of the early detection of subendocardial injuries. LGE-CMR [17] is the gold standard for accurately detecting irreversible myocardial injuries, and Gilles et al. found that the presence of subendocardial infarct-type LGE is an independent predictor of mortality in patients with AS [3,5]. It is noteworthy that patients with load-induced LVH commonly show a significant prevalence of chronic renal dysfunction. In a retrospective analysis of a series of 2408 patients undergoing surgical aortic-valve replacement, the prevalence of chronic kidney disease was reported to be ≈33.7%, including 7.2% with severe chronic kidney disease [7]. The pathogenesis of hypertension and that of chronic kidney disease are tightly intertwined; hypertension is both a complication of and a driver of kidney disease, and hypertension remains the second leading cause of end-stage renal disease [18]. The increasing concern about gadolinium-based contrast agents presents challenges to their use on these patients with severe renal dysfunction.

As a non-invasive technology without contrast, CMR-FT can be used in the quantitative evaluation of the early deformation of LV myocardium induced by scarring or fibrosis, which compromises the structural integrity of the myocardium and predisposes it to dysfunction [9]. In this study, we mainly compared the difference in CMR parameters between load-induced LVH with and without SILGE and explored the predictive value of CMR-FT-derived strain and strain parameters on SILGE in load-induced LVH. The higher NT-proBNP values in the SILGE^+^ group indicate that load-induced LVH with SILGE had a higher risk of heart failure compared to those without SILGE [19]: there is a strong correlation between the strain parameters and NT-proBNP.

The myocardium is a complex, three-dimensional structure, consisting of myocytes orientated in different directions with their own intrinsic contractile properties. CMR-FT can evaluate the motion of global myocardial fibers in different motion directions [20], in which RS describes the change in myocardial fibers from the epicardium to the endocardium, CS reflects the change in the myocardial fiber length at the short-axis level, and LS reflects the change in the long-axis muscle fiber length. In this study, strain and the strain rate in the SILGE^+^ group were lower than those in the SILGE^−^ group, indicating that SILGE signs are suggestive of systolic and diastolic impairments in load-induced LVH.

The typical characteristic of patients in this study was LV pressure overload. The data from research center 1 showed that the LVP in the SILGE^+^ group was higher than that in the SILGE^−^ group. Though all the indices of systolic function are altered by acute or chronic changes in the preload and afterload, and influenced by remodeling [11,12], our data analysis implies that the LVSP and LVEDP were well correlated with the GLS. Strain analysis showed that the strain parameters were sensitive markers of subclinical changes, reflecting SILGE in load-induced LVH, since it decreased with an endomyocardial injury. Moreover, throughout the whole study, multivariable logistic regression analysis indicated that the GLS was an independent predictive factor. The GLS showed the best diagnosis performance, which may be due to the longitudinal fibers located in the subendocardium that are more susceptible to ischemia and are, therefore, affected earlier in the ischemic cascade [21]. Since there was a higher correlation between the GLS and LVEDP compared to those of the other parameters, an alternative explanation could be that the longitudinal fibers exhibit a larger radius of curvature, rendering them more susceptible to the elevated stress from the left ventricle (LV) caused by a high filling pressure [22].

The complex anatomical orientation of myocardial fibers combined with the various factors influencing myocardial motions, such as contractility, interaction with the adjacent segments, and overall cardiac motion, can highlight the need to assess the regional myocardial function in the radial, circumferential, and longitudinal directions. Although our results show that the GLS had the best diagnostic and predictive performance for SILGE, which is akin to many research results, the radial and circumferential strains also reflect functional changes in the myocardial fibers in pressure-overload cardiomyopathy. The repeatability analysis of the strain parameters derived from using CMR-FT in this study also exhibited good intra- and inter-reader reproducibility.

The mechanisms of SILGE in load-induced LVH have not yet been elucidated. There are two main hypotheses: First, microvascular dysfunction from the compression is caused by the increased filling pressure and decreased vascular density due to secondary hypertrophy, leading to recurrent ischemia and fibrosis over time. The subendocardial layer is most vulnerable to extravascular compressive force impairment [4,23]. Second, the injured or compromised endocardium, resulting from the high pressure associated with high-turbulence flow, could play an important role in myocardial remodeling through both soluble signals and mechano-transduction [24,25]. Other histological studies found that in patients with load-induced cardiac hypertrophy, the endocardium is significantly thickened, and the degree of fibrosis is relatively severe, with a reduced fibrosis gradient from the endocardium to the middle of the myocardium [26]. The research on the mechanism of myocardial fibrosis caused by LV overload pressure suggested that most of the myocardial fibroblasts are derived from endocardial endothelial cells going through endothelial-to-mesenchymal transition [27,28,29]. When a contrast agent enters, the reduced ability of impaired or transited cells to wash out the contrast media creates conditions for the delayed enhancement of the subendocardium. The actual relationship between SILGE and the pathologic characteristics of the myocardium in load-induced LVH remains to be clarified, and further investigations are required.

## 5. Limitations

Several limitations should be kept in mind when interpreting our data. Although it was a multicenter study, the relationship between the LVP and LV strain parameters was only analyzed in research center 1 and cannot be verified in other research centers, so there may be some bias in the correlation analysis results. Neither T1 mapping nor ECV, both used to quantify myocardial fibrosis, were conducted in this study to compare the differences in myocardial fibrosis between the two groups of patients. In this study, endocardial biopsies were not performed on the patients. The actual relationship between the subendocardium-involved and the pathological characteristics of the myocardium in load-induced LVH remains to be clarified, and further investigations are required. There is a lack of follow-up and prognostic information on strain parameters in this study. In the future, long-term follow-ups on such patients should be made to validate and expand the findings of the present study.

## 6. Conclusions

Despite the load dependency of CMR-FT-derived strain analysis, the GLS demonstrated the accurate identification of SILGE. CMR-FT-derived strain analysis can potentially serve as a feasible alternative for detecting subendocardial involvement in patients with load-induced LVH who are contraindicated for LGE.

## Figures and Tables

**Table 1:** Clinical and CMR characteristics of population in load-induced LVH. patients with and without SILGE.

Variable	Overall *n* = 149	SILGE^+^ Group *n* = 56	SILGE^−^ Group *n* = 93	*p* Value
Clinical data				
Age, y	61 (21)	60 (16)	62 (25)	0.980
Male, sex	106 (71%)	45 (80%)	61 (66%)	0.054
Smoking history	51 (34%)	24 (43%)	27 (29%)	0.085
Hypertension	106 (71%)	36 (64%)	70 (75%)	0.152
Diabetes	36 (23%)	11 (20%)	25 (27%)	0.317
Coronary artery disease	0	0	0	-
NT-proBNP, (pg/mL)	1111.5 (2704.6)	1651.0 (2873.4)	956.5 (2029.4)	**0.010**
AS	66 (44%)	30 (54%)	36 (39%)	0.077
HHD	83 (56%)	26 (46%)	57 (61%)	0.077
CMR				
Cardiac function				
LVEDV, (mL)	183.89 (123.55)	184.42 (113.10)	178.93 (127.43)	0.207
LVESV, (mL)	103.54 (129.92)	111.52 (119.61)	91.76 (136.99)	0.144
LVSV, (mL)	75.06 (35.19)	77.81 (42.61)	77.09 ± 27.39	0.503
LVEF, (%)	45.45 (36.16)	42.21 ± 18.15	49.63 (38.56)	0.235
LVMI, (g/m^2^)	93.60 (38.83)	101.10 ± 30.72	91.96 ± 33.15	0.096
Strain and strain rate				
GRS, (%)	18.46 (16.37)	15.59 ± 7.45	21.17 (21.2)	**<0.001**
GCS, (%)	−13.15 ± 5.04	−11.09 ± 4.37	−14.39 ± 5.03	**<0.001**
GLS, (%)	−7.24 (5.54)	−5.01 ± 3.84	−9.40 ± 4.52	**<0.001**
GRSSR, (1/s)	1.19 (1.23)	1.12 ± 0.69	1.30 (1.44)	**0.012**
GCSSR, (1/s)	−0.71 (0.53)	−0.70 ± 0.37	−0.81 (0.50)	**0.040**
GLSSR, (1/s)	−0.50 (0.41)	−0.46 (0.43)	−0.55 (0.40)	0.123
GRDSR, (1/s)	−1.08 (1.00)	−0.92 (0.93)	−1.14 (1.09)	0.065
GCDSR, (1/s)	0.61 (0.37)	0.57 (0.38)	0.66 (0.40)	**0.048**
GLDSR, (1/s)	0.45 (0.29)	0.39 (0.34)	0.49 (0.29)	**0.019**

SILGE, subendocardium-involved late gadolinium enhancement; AS, aortic stenosis; HHD, hypertensive heart disease; LVEDV, left ventricular end-diastolic volume; LVESV, left ventricular end-systolic volume; LVSV, left ventricular stroke volume; LVEF, left ventricular ejection fraction; LVMI, left ventricular mass index; GRS, global radial strain; GCS, global circumferential strain; GLS, global longitudinal strain; GRSSR, global radial systolic strain rate; GCSSR, global circumferential systolic strain rate; GLSSR, global longitudinal systolic strain rate; GRDSR, global radial diastolic strain rate; GCDSR, global circumferential diastolic strain rate; GLDSR, global longitudinal diastolic strain rate. According to whether the statistics meet the normal distribution, continuous variables are expressed as mean ± standard deviation/median (interquartile range, IQR). Classification variables are expressed as *n* (%). *p* values of factors with bold values are less than 0.05.

**Table 2:** Logistic regression analysis in the prediction of SILGE.

Variable	Univariable Analysis		Multivariable Analysis	
	OR (95% CI)	*p* Value	OR (95% CI)	*p* Value
Age, y	1.005 (0.983–1.029)	0.643		
Male, sex	2.146 (0.978–4.709)	0.057		
Smoking history	1.833 (0.917–3.667)	0.087		
Hypertension	0.525 (0.253–1.091)	0.084		
Diabetes	0.665 (0.298–1.484)	0.319		
Disease type	1.827 (0.934–3.573)	0.078		
LVEDV, (mL)	1.002 (0.998–1.005)	0.304		
LVESV, (mL)	1.002 (0.998–1.005)	0.382		
LVSV, (mL)	1.004 (0.992–1.015)	0.539		
LVEF, (%)	0.989 (0.972–1.007)	0.227		
LVMI, (g/m^2^)	1.009 (0.998–1.019)	0.100		
GRS, (%)	0.928 (0.894–0.964)	**<0.001**		
GCS, (%)	1.153 (1.070–1.243)	**<0.001**		
GLS, (%)	1.325 (1.180–1.487)	**<0.001**	1.325 (1.180–1.487)	**<0.001**

CI, confidence interval; OR, odds ratio. *p* values of factors with bold values are less than 0.05.

**Table 3:** Intra- and inter-observer reproducibility for LV strain parameters.

	Overall	
	Intra-Observer	Inter-Observer
	ICC (95% CI)	ICC (95% CI)
GRS, %	0.992 (0.987–0.995)	0.988 (0.981–0.993)
GCS, %	0.988 (0.979–0.993)	0.975 (0.959–0.985)
GLS, %	0.983 (0.971–0.990)	0.970 (0.950–0.982)
	Research center 1	
	Intra-observer	Inter-observer
	ICC (95% CI)	ICC (95% CI)
GRS, %	0.994 (0.984–0.997)	0.990 (0.974–0.996)
GCS, %	0.982 (0.955–0.993)	0.963 (0.910–0.985)
GLS, %	0.988 (0.970–0.995)	0.975 (0.937–0.990)
	Research center 2	
	Intra-observer	Inter-observer
	ICC (95% CI)	ICC (95% CI)
GRS, %	0.990 (0.976–0.996)	0.982 (0.955–0.993)
GCS, %	0.989 (0.972–0.996)	0.974 (0.935–0.990)
GLS, %	0.983 (0.957–0.993)	0.968 (0.920–0.987)
	Research center 3	
	Intra-observer	Inter-observer
	ICC (95% CI)	ICC (95% CI)
GRS, %	0.992 (0.979–0.997)	0.989 (0.972–0.996)
GCS, %	0.990 (0.974–0.996)	0.980 (0.951–0.992)
GLS, %	0.984 (0.960–0.994)	0.976 (0.941–0.990)

ICC, intra-class correlation coefficient. ICC > 0.75 reflects high consistency.

## Data Availability

The data presented in this study are available on request from the corresponding author.

## Supplementary Materials

The following supporting information can be downloaded at: https://www.mdpi.com/article/10.3390/jcm12247543/s1. Table S1: Comparisons of LVP in load-induced LVH patients with and without SILGE in research center 1; Table S2: Correlation of LVP with strain parameters.
