# Supplementary material for: Feature-Tracking-Derived Strain Analysis for Identification of Subendocardium-Involved Late Gadolinium Enhancement in Load-Induced Left Ventricular Hypertrophy: A Multicenter Study of Cardiac Magnetic Resonance Data
Source: J Clin Med. 2023 Dec 7;12(24):7543. doi: 10.3390/jcm12247543 (PMC10744239; doi:10.3390/jcm12247543)
Supplement: Supplementary file 1 [file jcm-12-07543-s001.zip › jcm-2649321-supplementary.pdf]

Table S1. Comparisons of LVP in load-induced LVH patients with and without SILGE in Research Center 1

Variable	Overall n=73	SILGE+ group n=33	SILGE- group n=40	*P* value
LVSP, (mmHg)	165 (33)	175±25	163±22	**0.027**
LVEDP, (mmHg)	20 (6)	20 (5)	20±3	**0.026**

Abbreviations: LVSP, left ventricular systolic pressure; LVEDP, left ventricular end-diastolic pressure. *P*-values of factors with bold values are less than 0.05. Values are presented as mean ± SD or median (IQR).

Table S2. Correlation of LVP with strain parameters

Variable	LVSP		LVEDP	
	**r**	***P* value**	**r**	***P* value**
GRS	-0.156	0.187	-0.215	0.068
GCS	0.184	0.119	0.201	0.088
GLS	0.237	**0.044**	0.334	**0.004**

P-values of factors with bold values are less than 0.05.
